# Micro-CT of tracheal stenosis in trisomy 21

**DOI:** 10.1136/thoraxjnl-2018-212966

**Published:** 2019-02-02

**Authors:** Susan C Shelmerdine, Michael T Ashworth, Alistair D Calder, Nagarajan Muthialu, Owen J Arthurs

**Affiliations:** 1 Department of Clinical Radiology, Great Ormond Street Hospital for Children NHS Foundation Trust, London, UK; 2 Department of Histopathology, Great Ormond Street Hospital for Children NHS Foundation Trust, London, UK; 3 Department of Cardiothoracic Surgery, Great Ormond Street Hospital for Children NHS Foundation Trust, London, UK

**Keywords:** imaging/ct mri etc, thoracic surgery, airway epithelium, rare lung diseases

**Figure 1:**
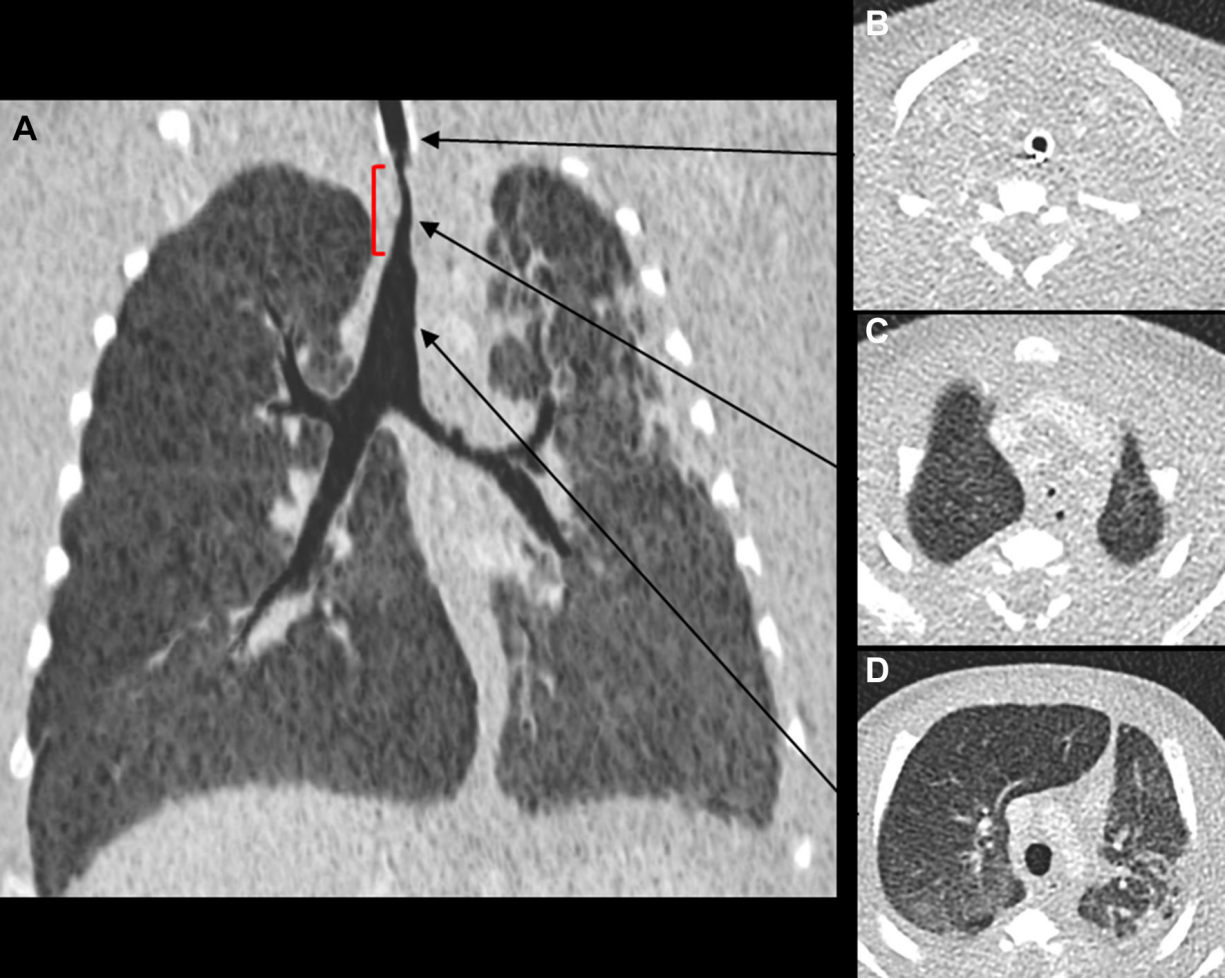
Antemortem CT thorax obtained 1 month prior to death. (A) A coronal 5 mm minimum intensity projection (miniP) image, viewed on lung windows, demonstrates the tight tracheal stenosis immediately distal to the endotracheal tube (red square bracket). Axial CT sections obtained (B) above, (C) at the level of the stenosis and (D) inferior to the stenosis show the relative narrowing of the trachea in relation to the intubated and non-stenosed sections (arrows).

A male infant with trisomy 21, born at 36 weeks' gestation, had care withdrawn at 2 months of age and was referred for postmortem investigations. The child had been ventilator dependent since the first week of life following surgery for intestinal perforation from necrotising enterocolitis. A CT thorax at 1 month of age demonstrated a tight tracheal stenosis, inferior to the tip of the endotracheal tube, with a luminal diameter of 1.2 mm over a length of 1 cm (figure 1). There were no associated cardiovascular or bronchial tree anomalies. Despite efforts to optimise the child’s condition for tracheal reconstruction, he was subsequently found to have an underlying immune deficit disorder with profound lymphopenia and continued to require iontropic support for haemodynamic compromise. After a further month of expectant clinical management without improvement, care was withdrawn.

The parents gave consent for a limited autopsy of the chest to further investigate the cause for the tracheal stenosis. Given the small size of the infant, and challenging nature of dissection, the excised trachea was imaged using microfocus CT (micro-CT). This technique uses multiple X-rays to create a high-resolution three-dimensional (3D) imaging dataset at a spatial resolution comparable to light microscopy (voxel size down to 1 micron). It has traditionally been used for non-destructive testing in the aerospace industry and in preclinical animal studies, but is gaining popularity with clinical applications including postmortem fetal imaging and breast pathological specimen analysis.^1^

The trachea was prepared using a solution of formalin and potassium iodide, and imaged at a resolution of 28 microns (0.028 mm). This was performed on a Med-X Alpha micro‐CT scanner (Nikon Metrology, Tring, UK) with a molybdenum target and no additional filtering. The tube current was 111 microamps, with energy of 90 kV, power of 10 W, gain of 24 dB. The exposure time was 354 ms, comprising 2294 projections, 4 X‐ray frames per projection, taking approximately 38 min to acquire. Scans were reconstructed using modified Feldkamp filtered back projection (with median filter kernel size 3) on proprietary software (CTPro3D; Nikon Metrology), and postprocessed using VG Studio MAX (Volume Graphics GmbH, Heidelberg, Germany).

**Figure 2:**
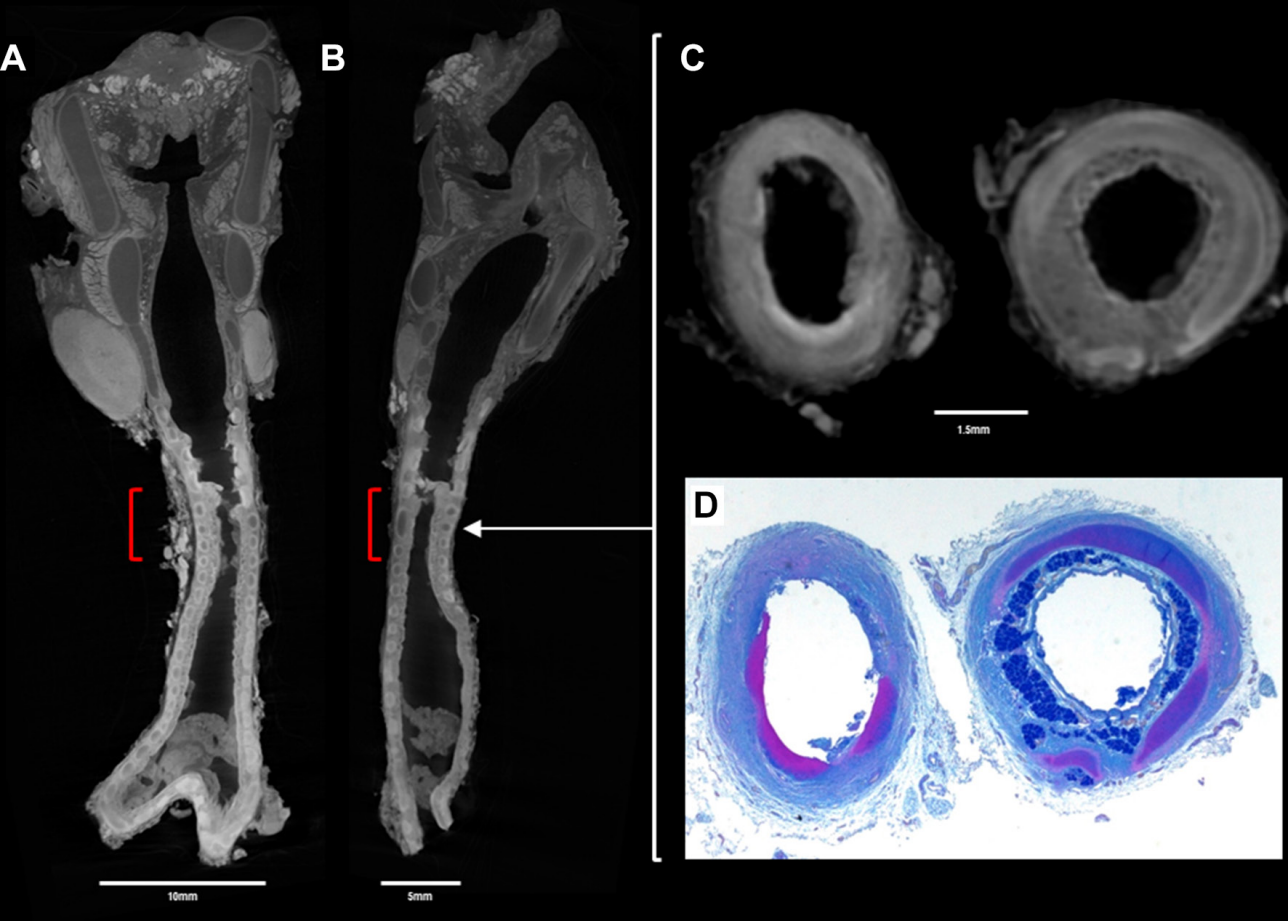
Postmortem (A) coronal and (B) sagittal micro-CT images, at 28 micron (0.028 mm) resolution of the excised trachea reveals a short segment, tight tracheal stenosis (red square bracket). Comparative axial sections through the trachea at the level of stenosis (white arrow) at both (C) micro-CT imaging and (D) histopathological examination (alcian blue and periodic acid-Schiff staining) show fibrosis of the tracheal wall and loss of mucous glands (left column images). The histological and matched micro-CT section images (right column) show cartilage (violet structure on histology, dark grey on micro-CT) encircling the majority of the tracheal circumference.

Imaging revealed multiple complete and bifid tracheal rings throughout the level of the stenosis, with associated areas of mucosal ulceration and tracheal wall fibrosis, the latter believed to result from prolonged ventilation and resultant tissue granulation. The findings were subsequently confirmed at histopathological dissection (figure 2).

Congenital tracheal stenosis is a rare airway anomaly estimated to occur in approximately 1 in 64 500 births, with several small case series reporting an association with trisomy 21.^2^ It is characterised by complete tracheal rings and, over 60% also have complex cardiac anomalies. The condition carries a significant mortality rate (as high as 77%), although with modern surgical reconstruction techniques, good survival rates (of approximately 88%) can be achieved.^3^

This case demonstrates the first documented usage of micro-CT technology on human tracheal tissue in this setting. The non-destructive nature of high-resolution imaging creates a digital dataset for 3D multiplanar visualisation of the whole specimen, at a comparable resolution as the histology. This technology may be used as an adjunct to plan pathological dissection, or as a replacement for specimen assessment in future research and clinical applications.^4^ The digital nature of the information lends itself to storage in a virtual repository which would be particularly useful should new clinical information be revealed and reassessment required at a later date.
